# First Molecular Typing of Tick-Derived *Coxiella burnetii* From Wildlife in South Korea: Surveillance and Genetic Characterization

**DOI:** 10.1155/tbed/2533438

**Published:** 2025-10-03

**Authors:** You-Jeong Lee, Su-Jin Chae, Beoul Kim, Hak Sub Shin, Sun Min Kwak, Hyesung Jeong, Suwoong Lee, Yong-Myung Kang, Dongmi Kwak, Min-Goo Seo

**Affiliations:** ^1^College of Veterinary Medicine and Institute for Veterinary Biomedical Science, Kyungpook National University, 80 Daehak-ro Buk-gu, Daegu 41566, Republic of Korea; ^2^Wildlife Disease Research Team, National Institute of Wildlife Disease Control and Prevention, 1 Songam-gil, Gwangju 62407, Republic of Korea

**Keywords:** *Coxiella burnetii*, *Coxiella*-like bacteria, MLVA, MST, One Health, tick

## Abstract

Q fever, caused by *Coxiella burnetii*, is a widespread zoonosis characterized by environmental persistence and a broad host range. Wildlife and their associated ticks are increasingly recognized as crucial elements in the ecology of this pathogen; however, molecular data from these reservoirs in South Korea remain scarce. From April to December 2024, 2747 ticks were collected from 297 wild animals across 16 regions of South Korea. Tick species were identified, and the spatial and temporal distributions of *C. burnetii* and *Coxiella*-like bacteria (CLB) were analyzed using molecular detection and minimum infection rate (MIR) estimation. Multilocus variable-number tandem-repeat analysis (MLVA) and multispacer sequence typing (MST) were used to genetically characterize *C. burnetii* detected in ticks. Four tick species were identified, with *Haemaphysalis longicornis* as the predominant species (84.6%). *C. burnetii* was detected at an overall MIR of 2.2%, varying by host species, tick species, region, and season. CLB was detected at a low MIR (0.5%), exclusively in ticks from Korean water deer. All *C. burnetii*-positive samples exhibited an MST77-like profile, and MLVA revealed a genotype closely related to those previously identified in placental samples from goats in France. This is the first report of this genotype in tick-derived samples from South Korea. These findings highlight the ecological importance of wildlife—particularly Korean water deer, raccoon dogs, roe deer, mountain rabbit, and badgers—in the maintenance and transmission of *C. burnetii*. The identification of genetically distinct *C. burnetii* genotypes and CLB underscores the need for ongoing molecular surveillance. This study supports a One Health approach to Q fever prevention and advances our understanding of the genetic diversity and transmission dynamics of the pathogen in wild ecosystems.

## 1. Introduction

Q fever is a globally distributed zoonosis caused by the obligate intracellular bacterium *Coxiella burnetii*. The bacterium primarily infects ruminant livestock, including sheep, goats, and cattle, which serve as the principal reservoirs for human transmission [1]. Humans are typically infected by inhaling aerosols contaminated with the pathogen or through direct contact with infected animals [2]. Although clinical symptoms in animals are often absent, infected hosts can shed *C. burnetii* in feces, urine, and milk, resulting in persistent environmental contamination [3].

Recent studies have increasingly examined the role of wild mammals and birds as additional reservoirs, highlighting the wildlife–tick interface as a key factor in the maintenance and circulation of *C. burnetii* [4]. Both hard and soft tick species are critical vectors of *C. burnetii*, with over 50 species identified as carriers [4]. Moreover, *Coxiella*-like bacteria (CLB), which are endosymbionts of ticks, are frequently detected and can be misidentified as *C. burnetii*, complicating molecular surveillance. Although direct transmission of *C. burnetii* to humans via ticks remains controversial, numerous studies have demonstrated the role of ticks in sustaining the circulation of *C. burnetii* and CLB within wildlife ecosystems [5].

Globally, *C. burnetii* has been detected across diverse wildlife taxa. In ungulates, postoutbreak surveillance in Spain reported prevalence rates of 7.0% in roe deer, 1.9% in wild boar, and 2.4% in red deer [6], suggesting circulation at the wildlife–livestock interface. In birds, molecular analyses of fecal samples revealed detection rates ranging from 0.6% to 13.7%, depending on the site [7]. In reptiles, a PCR survey of 605 wild turtles in the United States detected *C. burnetii* DNA in five individuals, suggesting that reptiles may act as incidental hosts [8]. In South Korea, *C. burnetii* and CLB have been detected in horses [9], as well as in ticks from horses [10], livestock, and wild animals [11]. In addition to bacterial pathogens, protozoan parasites have also been investigated in South Korea; for example, *Theileria orientalis* was detected in cattle and genetically characterized in a nationwide survey [12]. Unlike such protozoan studies, our work focuses on the bacterial zoonosis *C. burnetii* at the wildlife–tick interface.

Given the potential public health implications and the One Health relevance of Q fever, further investigation at the wildlife–tick interface is warranted. In this study, we targeted both the 16S rRNA gene [9] and the IS1111 gene [13]. The 16S rRNA gene enables differentiation of *C. burnetii* from CLB, whereas the IS1111 gene specifically detects *C. burnetii*. Previous studies have reported that the *C. burnetii* genome contains multiple IS1111 insertion sequences with variations in nucleotide sequence and PCR amplicon size. These allow classification into IS1111a, IS1111b, and IS1111c subtypes, which are useful for genotype comparison and tracing transmission pathways across regions [14]. In addition, IS1111 is present in multiple copies (7–110 per genome), which greatly enhances its sensitivity as a molecular detection target [15, 16]. This study aimed to assess the presence, spatial and temporal distribution, and genetic diversity—including multilocus variable-number tandem-repeat analysis (MLVA) and multispacer sequence typing (MST)—of *C. burnetii* and CLB in ticks collected from wild animals across South Korea. The findings are expected to improve understanding of pathogen circulation in wildlife ecosystems and contribute to evidence-based disease control strategies.

## 2. Materials and Methods

### 2.1. Ethical Approval

Ethical approval for all experimental procedures was granted by the Institutional Animal Care and Use Committee at Kyungpook National University (Approval Number KNU 2024-0407). Tick samples were obtained from wild animals captured by the Wildlife Management Institute, which also provided the specimens used in this study. Ticks attached to the wild animals were collected during routine capture operations. All procedures adhered to institutional ethical standards, and no additional handling was required. The collected ticks did not include endangered species.

### 2.2. Tick Collection and Species Identification

**Figure 1:**
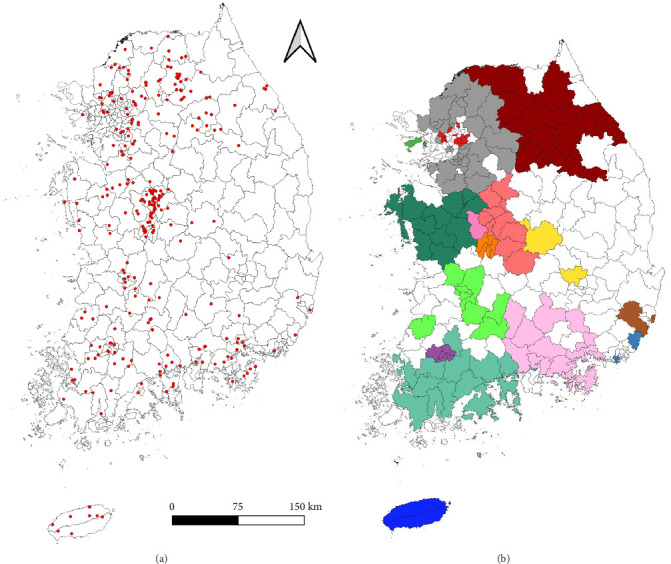
(a) Geospatial distribution of GPS coordinates for 297 wild animals sampled between April and November 2024. Each red dot denotes an individual sampling site. (b) Regional classification of the 16 administrative provinces in South Korea, grouped into northern, central, and southern regions. The northern region includes Seoul (SU), Incheon (IC), Gyeonggi (GG), and Gangwon (GW); the central region comprises Chungbuk (CB), Chungnam (CN), Sejong (SJ), Daejeon (DJ), Jeonbuk (JB), and Gyeongbuk (GB); and the southern region consists of Gyeongnam (GN), Busan (BS), Ulsan (US), Gwangju (GJ), Jeonnam (JN), and Jeju Island (JJ). Regions are color-coded as follows: GW (dark maroon), GG (gray), GN (light pink), GB (yellow), GJ (purple), DJ (orange), BS (navy blue), SU (bright red), SJ (reddish pink), US (brown), IC (teal), JN (forest green), JB (light green), JJ (deep blue), CN (green), and CB (bright red-orange).

Between April and November 2024, ticks were collected from wild animals across South Korea as part of a nationwide surveillance study. In total, 297 wild animals were captured, including Korean water deer (*n* = 202), raccoons (*n* = 59), roe deer (*n* = 26), badgers (*n* = 5), and single individuals of hedgehog, mountain rabbit, weasel, red squirrel, and Formosan sambar deer (Table 1, Figure 1a).

A total of 2747 ticks were collected: 1747 from Korean water deer, 576 from raccoons, 268 from roe deer, 76 from badgers, and smaller numbers from hedgehog (*n* = 3), mountain rabbit (*n* = 9), weasel (*n* = 3), red squirrel (*n* = 57), and Formosan sambar deer (*n* = 8).

The survey covered 16 administrative provinces in South Korea (Figure 1b). The number of ticks collected per region was: Gangwon ([GW] 308), Gyeonggi ([GG] 601), Gyeongnam ([GN] 160), Gyeongbuk ([GB] 36), Gwangju ([GJ] 74), Daejeon ([DJ] 96), Busan ([BS] 25), Seoul ([SU] 58), Sejong ([S], 14), Ulsan ([US] 29), Incheon ([IC] 6), Jeonnam ([JN] 245), Jeonbuk ([JB] 507), Jeju Island ([JJ] 147), Chungnam ([CN] 83), and Chungbuk ([CB] 358), totaling 2747 ticks.

After pooling, 2072 tick pools were prepared for molecular analysis. Pools were organized according to host species, tick species, developmental stage (adult, nymph, and larva), and collection site, with each pool containing only ticks meeting the same criteria. The number of ticks per pool ranged from one to three for nymphs, larvae, and adults. Initial identification was based on morphological features [8], followed by molecular confirmation to ensure accuracy. Samples were stored at −70°C until genomic DNA extraction.

### 2.3. DNA Extraction and Molecular Detection

Genomic DNA was extracted from tick samples using the Biniprep Pathogen DNA/RNA Kit (InvirusTech, GJ, Daegu) according to the manufacturer's instructions. PCR amplification was performed using the AccuPower PCR Premix Kit (Bioneer, Daejeon, South Korea). Nested PCR targeting the 16S rRNA gene was performed for the detection of *Coxiella burnetii* using the primer sets Cox16SF1/Cox16SR2 and Cox16SF2/Cox16SR2, generating a fragment of approximately 624 bp [9]. Additionally, PCR targeting the IS1111 gene was performed using the Trans 1/Trans 2 primer set, generating a fragment of approximately 687 bp [13]. Each tick was molecularly identified by amplifying the cytochrome c oxidase subunit I (cox1) gene from the mitochondria using designated primers [17]. Specifically, a *C. burnetii*-positive sample from Boer goats in our previous study in South Korea [18] was used as a positive control, while a CLB-positive sample from horses [9] was used as the positive control for CLB. A negative control (no DNA template) was included in each run to verify the absence of contamination.

### 2.4. MLVA Genotyping

MLVA genotyping was performed on PCR-positive samples using the AccuPower Hotstart PCR Premix Kit (Bioneer, South Korea). Six variable-number tandem-repeat markers (Ms23, Ms24, Ms27, Ms28, Ms33, and Ms34) were analyzed using specific primers and following the established protocol [19].

### 2.5. MST Genotyping

MST genotyping was performed using the AccuPower PCR Hotstart Premix Kit (Bioneer, South Korea). Ten spacers (Cox2, Cox5, Cox18, Cox20, Cox22, Cox37, Cox51, Cox56, Cox57, and Cox61) were analyzed using specific primers and following the established protocol [19].

### 2.6. DNA Sequencing and Phylogenetic Analysis

DNA sequencing of positive samples was performed at Macrogen (SU, South Korea) using specific primers. Comparative analysis was conducted by referencing nucleotide sequences from the GenBank database (NCBI). Sequence alignments were performed using CLUSTAL Omega (version 1.2.1) and further refined with BioEdit (version 7.2.5). Phylogenetic trees were constructed in MEGA (version 6.0) using the maximum likelihood method and the Kimura two-parameter distance model. The robustness of the phylogenetic tree was evaluated using bootstrap analysis with 1000 replicates.

### 2.7. Statistical Analysis

Statistical analyses were performed using GraphPad Prism (version 5.04, GraphPad Software Inc., La Jolla, CA, USA). Pearson's chi-square test was used to analyze contingency tables with more than two variables. A *p*-value ≤ 0.05 was considered statistically significant. A 95% confidence interval (CI) was calculated for each estimate.

## 3. Results

### 3.1. Tick Species Identification and Geographical Distribution

**Figure 2:**
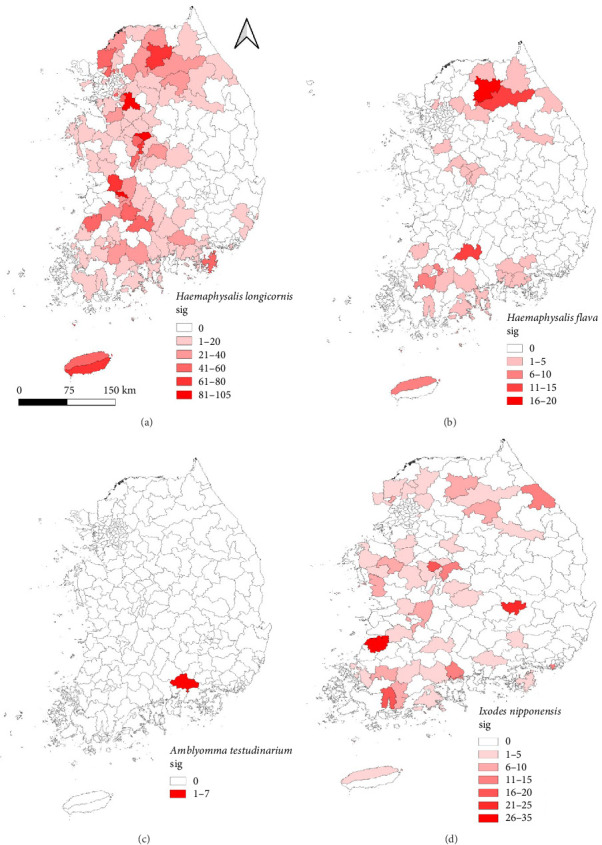
Geographical distribution of four tick species collected from wild animals at 16 sampling locations across South Korea in 2024. (a) *Haemaphysalis longicornis*, (b) *H. flava*, (c) *Amblyomma testudinarium*, and (d) *Ixodes nipponensis*. Map shading indicates the number of ticks collected, with darker colors representing higher densities.

A total of 2747 ticks were collected and grouped into 2072 pools for analysis. Four species were identified: *Haemaphysalis longicornis* (2325 ticks; 1691 pools; Figure 2a), *Haemaphysalis flava* (120 ticks; 118 pools; Figure 2b), *Amblyomma testudinarium* (7 ticks; 7 pools; Figure 2c), and *Ixodes nipponensis* (295 ticks; 256 pools; Figure 2d) (Table 2).

Tick pools were collected across 16 regions: GW (252), GG (430), GN (126), GB (24), GJ (50), DJ (52), BS (21), SL (37), SJ (14), US (17), IC (6), JN (193), JB (386), JJ (118), CN (78), and CB (268). Among the 2072 tick pools, 1462 were adult pools, 518 were nymph pools, and 92 were larval pools.

### 3.2. Wildlife Host Distribution

A total of 297 wild animal specimens were collected across 16 administrative regions in South Korea (Table 1). The collection sites were categorized by region: the northern region included SL (*n* = 5), IC (*n* = 2), GG (*n* = 60), and GW (*n* = 48); the central region included CB (*n* = 46), CN (*n* = 16), SJ (*n* = 4), DJ (*n* = 14), JB (*n* = 24), and GB (*n* = 3); and the southern region included GN (*n* = 25), BS (*n* = 2), US (*n* = 2), GJ (*n* = 10), JN (*n* = 28), and JJ (*n* = 8). Geographic coordinates for all specimens were recorded on-site using handheld GPS devices. A spatial map of the 297 sampling locations was generated (Figure 1a).

### 3.3. Prevalence of CLB and *C. burnetii* by Tick Species

The overall positivity rates for CLB and *C. burnetii* were 0.4% (8/2072 pools) and 2.9% (60/2072 pools), respectively (Table 2). The corresponding minimum infection rates (MIRs) were 0.3% (8/2747) for CLB and 2.2% (60/2747) for *C. burnetii*. Detection by tick species was as follows: in *I. nipponensis*, CLB was not detected, while *C. burnetii* was identified in 0.4% of pools (1/256; 95% CI: 0%–1.2%), with a MIR of 0.3% (1/295). In *A. testudinarium*, neither CLB nor *C. burnetii* was detected. In *H. flava*, CLB was not detected, whereas *C. burnetii* was found in 3.4% of pools (4/118; 95% CI: 0.1%–6.7%), with a MIR of 3.3% (4/120). In *H. longicornis*, CLB was detected in 0.5% of pools (8/1691; 95% CI: 0%–0.8%), and *C. burnetii* in 3.3% of pools (55/1691; 95% CI: 2.4%–4.1%), corresponding to MIRs of 0.3% (8/2325) for CLB and 2.4% (55/2325) for *C. burnetii*.

### 3.4. Prevalence of CLB and *C. burnetii* by Region

**Figure 3:**
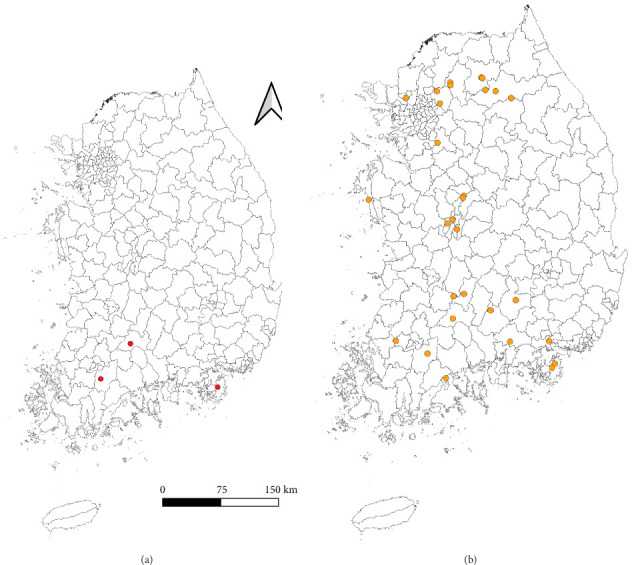
Geographical distribution of regions where tick samples tested positive for (a) *Coxiella*-like bacteria and (b) *Coxiella burnetii*. Each dot indicates the sampling location of an individual wild animal from which ticks were collected.

The regional distribution of CLB and *C. burnetii* positivity is summarized in Table 2 and Figure 3. The detection rates were as follows: GW: CLB not detected; *C. burnetii* detected in 6.7% of pools (17/252; 95% CI: 3.6–9.8). GG: CLB not detected; *C. burnetii* in 1.6% of pools (7/430; 95% CI: 0.4–2.8). GN: CLB in 0.8% of pools (1/126; 95% CI: 0–2.3); *C. burnetii* in 8.7% of pools (11/126; 95% CI: 3.8–13.7). GJ: CLB not detected; *C. burnetii* in 10.0% of pools (5/50; 95% CI: 1.7–18.3). DJ: CLB not detected; *C. burnetii* in 13.5% of pools (7/52; 95% CI: 4.2–22.7). JN: CLB in 0.5% of pools (1/193; 95% CI: 0–1.5); *C. burnetii* in 1.0% of pools (2/193; 95% CI: 0–2.5). JB: CLB in 1.6% of pools (6/386; 95% CI: 0.3–2.8); *C. burnetii* in 2.1% of pools (8/386; 95% CI: 0.7–3.5). CB: CLB not detected; *C. burnetii* in 0.7% of pools (2/268; 95% CI: 0–1.8). In the remaining surveyed regions (GB, BS, SL, US, IC, JJ, and CN), neither CLB nor *C. burnetii* was detected.

### 3.5. Prevalence of CLB and *C. burnetii* by Month

**Figure 4:**
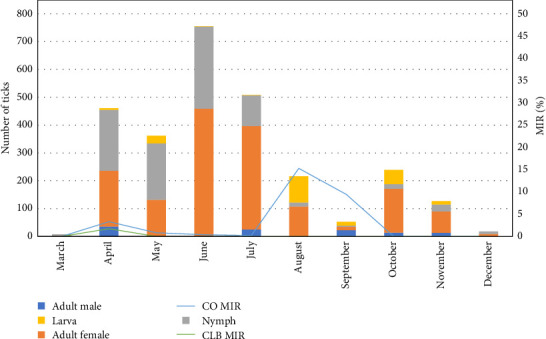
Monthly distribution of tick populations and MIR of *Coxiella burnetii* (CO) and CLB in ticks collected from wildlife across South Korea between April and December 2024.

The number of ticks collected each month was as follows (Table 3, Figure 4): March (*n* = 8), April (*n* = 462), May (*n* = 362), June (*n* = 754), July (*n* = 508), August (*n* = 216), September (*n* = 53), October (*n* = 239), November (*n* = 127), and December (*n* = 18), totaling 2747 ticks. The corresponding tick pool counts were: March (*n* = 5), April (*n* = 364), May (*n* = 246), June (*n* = 574), July (*n* = 388), August (*n* = 153), September (*n* = 32), October (*n* = 196), November (*n* = 102), and December (*n* = 12), for a total of 2047 pools.

For *I. nipponensis*, *C. burnetii* was detected only in April, with a prevalence of 1.4% (1/69 pools; 95% CI: 0–4.3); CLB was not detected. In *A. testudinarium*, neither *C. burnetii* nor CLB was detected in any month. For *H. flava*, CLB was negative throughout, but *C. burnetii* was detected in April with a prevalence of 10.0% (3/30 pools; 95% CI: 0–20.7). In *H. longicornis*, CLB was detected in April at 3.1% (8/258 pools; 95% CI: 1.0–5.2), and *C. burnetii* displayed its highest monthly prevalence in August at 21.2% (32/151 pools; 95% CI: 14.7–27.7).

Based on monthly MIR analysis, *C. burnetii* had its highest infection rate in August at 15.3% (33/216; 95% CI: 10.5–20.1), followed by September (9.4%, 5/53; 95% CI: 1.6–17.3), April (3.3%, 15/461; 95% CI: 1.6–4.9), May (0.8%, 3/362; 95% CI: 0–1.8), June (0.4%, 3/755; 95% CI: 0–0.8), and July (0.2%, 1/508; 95% CI: 0–0.6). No *C. burnetii* was detected in March or from October through December. For CLB, the highest MIR was observed in April at 1.7% (8/461; 95% CI: 0.5–2.9), with no detections recorded in March or from May to December.

### 3.6. Prevalence of CLB and *C. burnetii* by Animal Species

Tick pools were collected from the following wild animal species (Table 4): Korean water deer (*n* = 1369), hedgehog (*n* = 2), raccoon dog (*n* = 386), roe deer (*n* = 234), mountain rabbit (*n* = 4), badger (*n* = 45), weasel (*n* = 3), red squirrel (*n* = 21), and Formosan sambar deer (*n* = 8).

CLB was detected exclusively in ticks from Korean water deer, with a prevalence of 0.6% (8/1369 pools; 95% CI: 0.2–1.0). No CLB was detected in ticks from any other host species. A chi-square test revealed significant differences in *C. burnetii* prevalence among host species (*χ*^2^ = 151.340, df = 4, *p* < 0.0001). The prevalence was highest in ticks from mountain rabbits (100%, 4/4 pools), followed by badgers (11.1%, 5/45 pools; 95% CI: 1.9–20.3), raccoon dogs (4.7%, 18/386 pools; 95% CI: 2.6–6.8), Korean water deer (2.2%, 30/1369 pools; 95% CI: 1.4–3.0), and roe deer (1.3%, 3/234 pools; 95% CI: 0–2.7). No *C. burnetii* was detected in ticks from hedgehogs, weasels, red squirrels, or Formosan sambar deer. Although ticks from mountain rabbits showed the highest prevalence, this estimate is based on a very small sample size (*n* = 4 pools), which limits the strength of statistical interpretation. These results indicate that host species differ in their contribution to the maintenance of *C. burnetii* within wildlife–tick ecosystems.

### 3.7. MLVA Genotyping Results

MLVA genotyping was performed on all *C. burnetii* PCR-positive samples. A single genotype was identified, displaying a uniform tandem-repeat pattern of 4–11–4–5–8–2 at the six loci (MS23, MS24, MS27, MS28, MS33, and MS34). No variation was observed, and this profile was designated as the representative MLVA genotype for this study. Comparison with the MLVA database (https://microbesgenotyping.i2bc.paris-saclay.fr/databases) showed that this genotype closely matched a sequence previously identified in a human heart sample from France.

### 3.8. MST Genotyping Results

**Figure 5:**
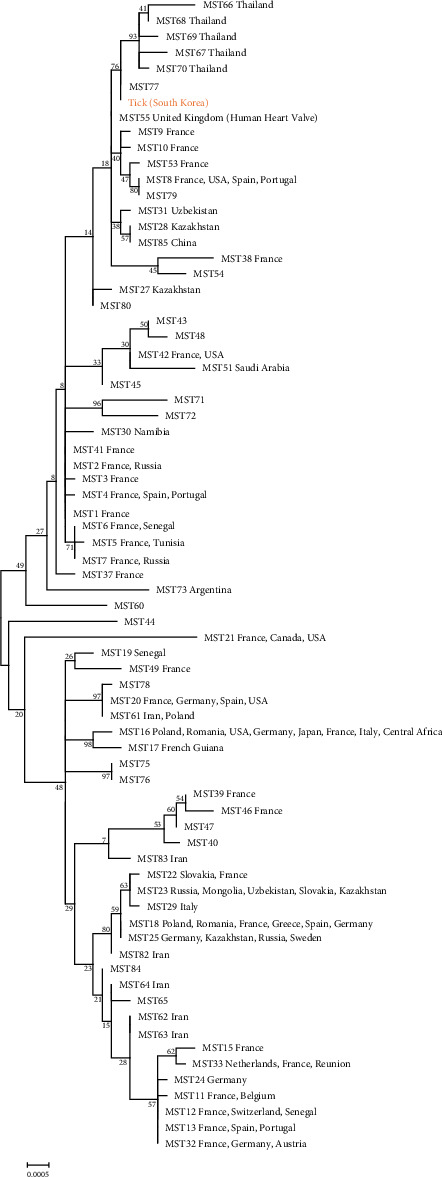
A maximum likelihood phylogenetic tree illustrating genetic relationships among different MST groups was generated using MEGA6 software. The analysis included *C. burnetii* sequences from tick samples and representative sequences from other MST groups, applying the Kimura 2-parameter model with a gamma (γ) distribution and 1000 bootstrap iterations.

MST genotyping was performed on all *C. burnetii*-positive samples using 10 genetic markers (Cox2, Cox5, Cox18, Cox20, Cox22, Cox37, Cox51, Cox56, Cox57, and Cox61). A single genotype was identified across all samples and is presented in Figure 5. The tick samples exhibited a spacer-type profile of 5–4–9–5–8–5–14–3–4–6, which closely corresponded to MST77, a genotype for which metadata are currently unavailable. Genotypes were determined using the MST database and the BLAST tool (v. 2.14.1) available at https://ifr48.timone.univ-mrs.fr/mst/coxiella_burnetii/blast.html. The resulting MST sequences were also compared with publicly available reference sequences in the NCBI GenBank database via BLAST. This comparison revealed high similarity to *C. burnetii* sequences (accession numbers CP107268 and CP107247) previously isolated from human blood samples collected in Cheongju and Iksan, South Korea, in 2016.

### 3.9. Molecular and Phylogenetic Analyses

**Figure 6:**
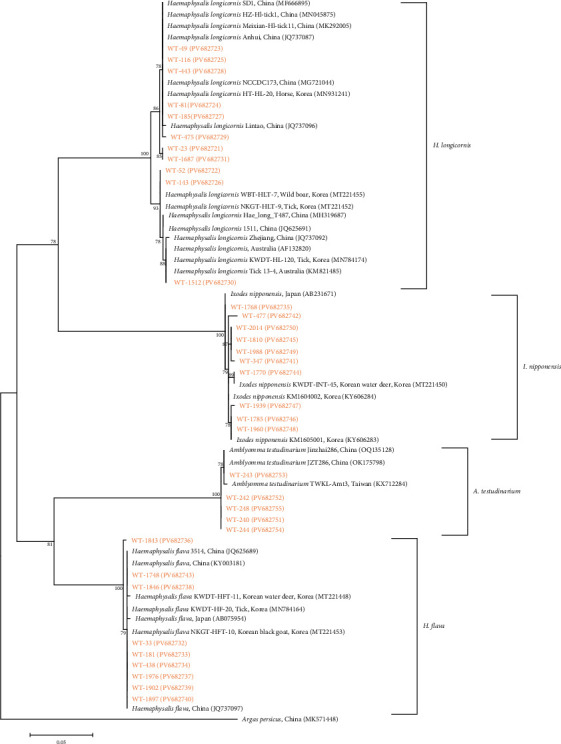
Molecular identification of tick species was performed by phylogenetic analysis using the maximum likelihood method based on the mitochondrial cytochrome c oxidase subunit I (cox1) gene. Sequences analyzed in this study are marked in orange. Nucleotide accession numbers from GenBank are displayed alongside the corresponding species names and countries of origin. Numbers on branches represent bootstrap support values based on 1000 replicates, and the scale bar indicates genetic distance.

Eleven representative *H. longicornis* cox1 sequences obtained in this study exhibited 98.1%–100% identity among themselves (Figure 6), along with 92.4%–100% identity relative to previously published *H. longicornis* cox1 sequences in GenBank. Ten representative *I. nipponensis* cox1 sequences showed 99.2%–100% identity within the group and 98.9%–100% identity with reference sequences in GenBank. Nine *H. flava* cox1 sequences displayed 99.5%–100% internal identity and 98.7%–100% identity with GenBank records. Five *A. testudinarium* cox1 sequences exhibited 96.5%–100% identity among themselves and 94.5%–96.6% identity with corresponding sequences in GenBank. All cox1 sequences generated in this study were deposited in GenBank under accession numbers PV682721–PV682755.

**Figure 7:**
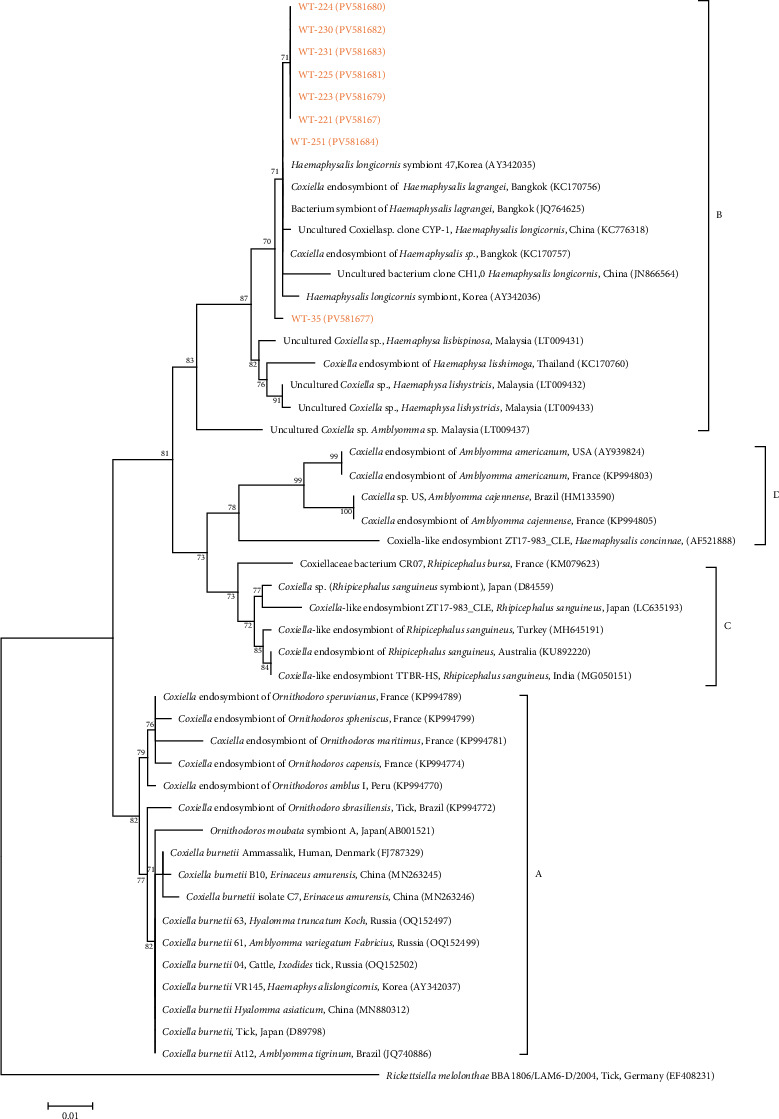
A phylogenetic tree was constructed using 16S rRNA sequences of *C. burnetii* with the maximum likelihood method. Sequences analyzed in this study are highlighted in orange. GenBank accession numbers for reference sequences are shown alongside their sequence names and countries of origin. *Rickettsiella melolonthae* was used as the outgroup. Bootstrap support values (1000 replicates) are indicated on the branches, and the scale bar denotes phylogenetic distance. *Coxiella* species are classified into four distinct clades, designated A through D.

Eight representative CLB 16S rRNA gene sequences showed 99.5%–100% identity among themselves and 95.5%–100% identity with previously registered CLB clade B sequences in GenBank (Figure 7). These sequences were submitted to GenBank under accession numbers PV581677–PV581684.

**Figure 8:**
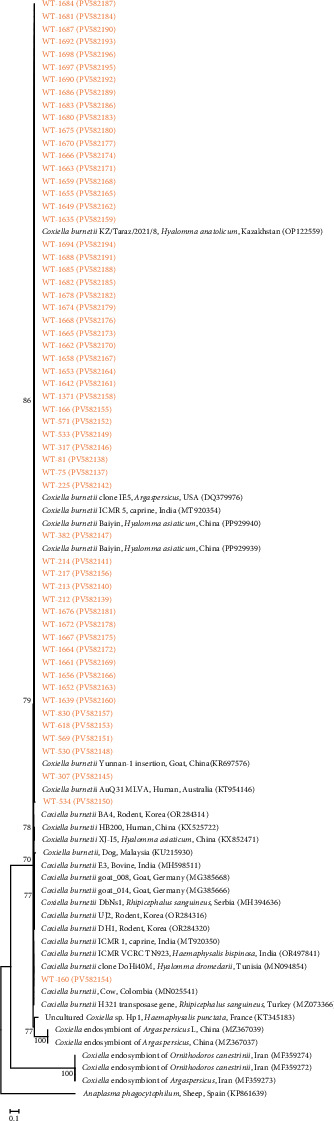
A phylogenetic tree was constructed using IS1111 sequences of *C. burnetii* with the maximum likelihood method. Sequences analyzed in this study are highlighted in orange. GenBank accession numbers for reference sequences are provided alongside their sequence names. *Anaplasma phagocytophilum* served as the outgroup. Bootstrap support values (1000 replicates) are shown on the branches, and the scale bar indicates phylogenetic distance.

Additionally, 58 representative *C. burnetii* IS1111 transposase gene sequences exhibited 99.5%–100% identity among themselves and 94.6%–100% identity compared to GenBank records (Figure 8). All IS1111 sequences generated in this study were deposited in GenBank under accession numbers PV582137–PV582196.

## 4. Discussion

Ticks were collected from wild animals across 16 regions in South Korea. Four tick species were identified, with *H. longicornis* being the most prevalent (84.6%), followed by *I. nipponensis* (10.7%), *H. flava* (4.4%), and *A. testudinarium* (0.3%). These findings are consistent with previous studies identifying *H. longicornis* as the dominant tick species in South Korea, with prevalence rates exceeding 90% [20]. *A. testudinarium* was detected only in the GN region, consistent with prior studies suggesting this species is primarily distributed in southern provinces, such as JN, JB, and GN, and rarely observed in central regions [21]. However, other research has reported the presence of *A. testudinarium* in DJ, a central region of South Korea [22]. These results indicate that ecological and environmental factors, including recent global warming, may influence tick distribution. The range of *A. testudinarium* may have expanded northward compared to historical patterns.

The overall MIR of *C. burnetii* was 2.2%. Regionally, MIR values were highest in DJ (7.3%), GN (6.9%), GJ (6.8%), and GW (5.5%), followed by JB (1.6%), GG (1.2%), CN (1.2%), JN (0.8%), and CB (0.6%). By tick species, *C. burnetii* DNA was detected in *H. flava* (3.3%) and *H. longicornis* (2.4%), whereas no PCR-positive pools were identified in *A. testudinarium* or *I. nipponensis*. These findings indicate a heterogeneous spatial distribution of *C. burnetii*, likely reflecting regional variation in host availability, landscape features, and tick density. Future studies should incorporate environmental variables and host surveillance data to further elucidate these ecological patterns. The overall MIR for CLB was 0.3%, and CLB was detected only in *H. longicornis*. This rate is notably lower than expected, as CLBs are typically considered common endosymbionts in ticks. The limited detection may be attributable to ecological and microbiological factors, including microbial competition, host specificity, and environmental influences. Recently, a novel *Coxiella*-like species, *Candidatus Coxiella massiliensis*, was identified in tissue samples from patients presenting with tick bites and associated skin lesions or cervical lymphadenopathy. This emerging microorganism may represent one of the etiological agents responsible for such clinical syndromes. Further studies are needed to clarify whether *Ca*. *C. massiliensis* exemplifies the evolutionary transition from a tick symbiont to a human pathogen, underscoring the potential public health relevance of CLB beyond their role as tick endosymbionts [4].

Seasonal changes in humidity and temperature strongly influence tick development, survival, and distribution, thereby shaping the risk of tick-borne pathogen transmission. In this study, tick collection data showed that tick activity began to increase in April, reached its peak during summer, and declined after September. Adult females were most abundant in June and July, followed by nymphs. After August, the number of adult females decreased while larval populations rose. These results are consistent with previous studies in South Korea, which report that nymphs are predominant from April to June, adult ticks peak in June and July, and larvae are most prevalent between August and September [20, 22, 23]. This seasonal distribution reflects the typical tick life cycle: adult females lay eggs in summer, and larvae emerge and develop from late summer into early autumn. Such temporal patterns underscore the influence of climatic and ecological variables on tick population dynamics and pathogen transmission potential. Future research should incorporate fine-scale meteorological and ecological data to better elucidate these relationships.

Ticks in this study were collected from various wild animals, with Korean water deer yielding the highest numbers (*n* = 1369), followed by raccoon dogs (*n* = 386) and roe deer (*n* = 234). These findings are consistent with previous studies, including reports of over 6600 ticks collected from 39 Korean water deer between 2019 and 2020 [24] and average adult tick burdens of 16–25 per individual in two separate studies [25, 26]. The high tick burden in Korean water deer may result from their broad geographic range and ecological adaptability, as they inhabit wetlands, grasslands, and forests [27, 28], all favorable for tick exposure. Raccoon dogs also exhibited substantial tick infestations, consistent with earlier findings in South Korea that reported 114 ticks from three individuals [29]. These observations suggest that both Korean water deer and raccoon dogs are key hosts at the wildlife–tick interface and may play important roles in maintaining and circulating tick-borne pathogens in the environment.

Given the ecological role of wild animals as potential reservoirs, this study assessed the prevalence of *C. burnetii* in ticks collected from wildlife inhabiting areas where wild and domestic animals coexist. In South Korea, *C. burnetii* infections have been reported over the past decade in various livestock species, including cattle, pigs, horses, and both native Korean and Boer goats [18, 30–33]. Additionally, *C. burnetii* was detected in 1.2% (5/5644) of ticks collected from livestock and wild animals in 2019 [11], and a prevalence rate of 14.6% (142/975) was reported in wild boars [34], underscoring the reservoir potential of wildlife. In this study, although sample sizes varied by species, the MIR of *C. burnetii* was estimated at 44.4% in mountain rabbit, 6.6% in badgers, 3.1% in raccoon dogs, 1.7% in Korean water deer, and 1.1% in roe deer. These results indicate that wild animals may serve as important reservoirs or contributors to the environmental transmission of *C. burnetii*, emphasizing the need for ongoing surveillance and targeted risk mitigation strategies.

Beyond *C. burnetii*, ticks also harbor a diverse range of CLB, which are phylogenetically distinct but closely related endosymbionts. Based on 16S rRNA gene analysis, members of the *Coxiella* genus are classified into four major clades (A–D), with *C. burnetii* in clade A [5]. In South Korea, previous studies have identified CLB belonging to clade B and novel genetic lineages [9, 10], indicating considerable genetic diversity among local CLB populations. For example, one study reported 1.3% positivity for *C. burnetii* by ELISA and 0.7% for CLB by PCR in horse samples [9], while another study found CLB in 52.4% of 213 ticks collected from horses in JJ, all assigned to clade B [10]. In the current study, CLB was detected exclusively in ticks collected from Korean water deer (8/1747; MIR 0.5%) and was identified as belonging to clade B. Although this detection rate is lower than previously reported rates, it suggests that Korean water deer may serve as potential vertebrate hosts in the natural transmission cycle of CLB. CLB have been localized to the salivary glands of ticks, raising concerns about possible transmission to vertebrates during blood feeding [5]. While the pathogenicity and zoonotic potential of CLB remain undetermined, their phylogenetic proximity to *C. burnetii* and widespread occurrence in ticks feeding on both wild and domestic animals underscore the need for further research. However, reports of CLB detection in vertebrate hosts, aside from transmission via tick vectors, remain extremely limited, and systematic studies on their epidemiology and pathogenicity are lacking. These findings highlight the need for ongoing molecular surveillance and comparative genomic studies across a wider range of tick vectors and host species. A One Health approach—integrating wildlife, livestock, and human health perspectives—is essential to understand the ecological behavior, host specificity, and potential health risks posed by CLB. In this study, the 16S rRNA gene was used to differentiate *C. burnetii* from CLB, and the IS1111 gene was selected as the primary target for *C. burnetii* detection. IS1111 is a multicopy element (7–110 copies per genome) and has been shown to provide higher detection sensitivity than single-copy genes, including isocitrate dehydrogenase (*icd*) gene [16]. Consequently, IS1111-based PCR/qPCR assays have been widely applied for the detection of *C. burnetii* from diverse sample types, such as livestock, wildlife, and environmental samples. Although the single-copy *icd* gene was not included in this study, future research will incorporate *icd* in parallel with IS1111 to further improve the accuracy of genotyping and source tracking.

We analyzed the genetic characteristics of *C. burnetii* detected in ticks from South Korea using MLVA and MST genotyping. MLVA was performed at six loci (MS23, MS24, MS27, MS28, MS33, and MS34), resulting in the tandem-repeat profile 4–11–4–5–8–2. Comparison with the MLVA database indicated that this genotype closely matches Cb#011 and Cb#012, previously identified in placental samples from aborted goats in France. Earlier studies from South Korea detected genotype 4–18–3–5–8–2 in Boer goats [18], which closely resembles a genotype previously found in a human endocarditis case in France. Another genotype, 6–13–2–7–9–10, identified in cattle [19], has also been reported in several European countries, including the Netherlands, France, Spain, and Poland, suggesting potential international dissemination or conserved transmission routes. In Yunnan Province, China, *C. burnetii* was identified in 3.1% of 516 *Rhipicephalus microplus* ticks collected from cattle. MLVA analysis of 10 positive samples, using the same six VNTR markers, produced a uniform profile of 9–27–4–6–9–5. This genotype has also been reported in humans and ticks from the United States, France, and Canada [35], suggesting the circulation of genetically related strains across regions and a potential role for tick vectors in their dissemination.

MST analysis of *C. burnetii* in this study showed that all tick-derived sequences had an identical spacer combination of 5–4–9–5–8–5–14–3–4–6. Comparison with the MST database indicated a high similarity to genotype MST77, although specific host data for this genotype are not available. In South Korea, MST analysis of tick-derived *C. burnetii* has not previously been reported, although MST typing of livestock-derived isolates has been conducted. Previous studies identified an MST61-related genotype in cattle, which was also detected in cow milk samples from Poland and Iran and is closely related to MST20—commonly found in France, Germany, the United States, and Spain [19]. In Boer goats, MST55 and MST80 genotypes were reported, with MST55 similar to a human-derived strain from the United Kingdom [18]. Internationally, MST analysis of tick-derived *C. burnetii* has revealed considerable genotype diversity. In Gansu Province, China, 46.9% (90/192) of *Hyalomma marginatum* ticks tested positive, and a novel genotype, MST85, was identified [36]. In Ethiopia's Oromia region, *C. burnetii* was detected in 6.4% of 842 ticks, with MST18 found in southeastern areas and MST20 in central areas [37]. Collectively, these findings demonstrate substantial genetic diversity of *C. burnetii* in tick populations worldwide. The observed variability likely reflects differences in ecological context, tick vector species, and regional host composition, underscoring the importance of ongoing molecular surveillance in diverse geographic settings. Prior studies have established a foundation for understanding the genetic characteristics of *C. burnetii* and for tracing molecular epidemiological links between domestic and international strains. Within this framework, the present study provides the first MLVA and MST analysis of tick-derived *C. burnetii* in South Korea. The findings demonstrate the presence of distinct genotypes circulating in local tick populations, suggesting the involvement of previously unrecognized transmission pathways and the existence of regionally maintained lineages.

## 5. Conclusions

This study presents a molecular epidemiological investigation of *C. burnetii* and CLB in ticks collected from wildlife in South Korea, including the nation's first MLVA and MST genotyping of tick-derived *C. burnetii*. The results offer new insights into the distribution, host associations, and genetic diversity of these pathogens at the wildlife–tick interface. *H. longicornis* was the predominant tick species and the detection of *C. burnetii* in ticks collected from Korean water deer, raccoon dogs, roe deer, mountain rabbit, and badgers suggests that these wild mammals may serve as important ecological reservoirs. The variation in the MIR of *C. burnetii* across tick species, host animals, geographic regions, and seasons underscores the multifactorial nature of its transmission dynamics. Although CLB showed low MIR, their presence in ticks and wild herbivores indicates potential ecological importance and warrants further investigation, particularly given their phylogenetic proximity to *C. burnetii*. Notably, all MST-typed *C. burnetii*-positive samples showed an identical genotype (MST77-like), suggesting the circulation of a localized lineage potentially adapted to South Korean tick populations. Overall, these findings underscore the need for continued molecular surveillance of *C. burnetii* and CLB within a One Health framework. Ongoing research is essential to clarify their pathogenicity, host specificity, and environmental persistence, which are critical for designing effective public health strategies for Q fever prevention and control. Therefore, the findings of this study suggest that ticks play a key role in sustaining the natural cycle of *C. burnetii* in wildlife ecosystems, highlighting their significance in the broader epidemiology of Q fever.

## Figures and Tables

**Table 1:** Numbers of wild animal hosts by species and region used for tick collection.

Region^a^	Korean water deer	Hedgehog	Raccoon dog	Roe deer	Mountain rabbit	Badger	Weasel	Red squirrel	Formosan sambar deer	Total
GW	18	0	14	14	1	0	1	0	0	48
GG	38	0	19	1	0	1	0	1	0	60
GN	21	0	3	0	0	1	0	0	0	25
GB	3	0	0	0	0	0	0	0	0	3
GJ	6	0	3	0	0	1	0	0	0	10
DJ	14	0	0	0	0	0	0	0	0	14
BS	2	0	0	0	0	0	0	0	0	2
SU	1	0	4	0	0	0	0	0	0	5
SJ	3	0	1	0	0	0	0	0	0	4
US	2	0	0	0	0	0	0	0	0	2
IC	2	0	0	0	0	0	0	0	0	2
JN	21	0	5	0	0	1	0	0	1	28
JB	17	0	3	3	0	1	0	0	0	24
JJ	0	0	0	8	0	0	0	0	0	8
CN	12	0	4	0	0	0	0	0	0	16
CB	42	1	3	0	0	0	0	0	0	46
Total	202	1	59	26	1	5	1	1	1	297

^a^16 regions in South Korea: Gangwon (GW), Gyeonggi (GG), Gyeongnam (GN), Gyeongbuk (GB), Gwangju (GJ), Daejeon (DJ), Busan (BS), Seoul (SU), Sejong (SJ), Ulsan (US), Incheon (IC), Jeonnam (JN), Jeonbuk (JB), Jeju Island (JJ), Chungnam (CN), and Chungbuk (CB).

**Table 2:** Detection of *Coxiella*-like bacteria and *Coxiella burnetii* by tick species, developmental stage, and region, including minimum infection rates.

Species	Stage	Tested ticks (pool)	Number of positive tick pools/Number of tick pools tested by region^a^																																		MIR^b^	
			GW		GG		GN		GB		GJ		DJ		BS		SL		SJ		US		IC		JN		JB		JJ		CN		CB		Total			
			CLB	CO	CLB	CO	CLB	CO	CLB	CO	CLB	CO	CLB	CO	CLB	CO	CLB	CO	CLB	CO	CLB	CO	CLB	CO	CLB	CO	CLB	CO	CLB	CO	CLB	CO	CLB	CO	CLB	CO	CLB	CO
*Ixodes nipponensis*	Adult male	33 (21)	0/4	0/4	0	0	0	0	0/3	0/3	0	0	0	0	0	0	0	0	0	0	0	0	0	0	0/6	0/6	0/6	0/6	0	0	0	0	0/2	0/2	0/21	0/21	0	0
	Adult female	194 (194)	0/21	0/21	0/29	1/29	0/10	0/10	0/13	0/13	0/3	0/3	0/1	0/1	0/6	0/6	0	0	0/1	0/1	0	0	0	0	0/46	0/46	0/12	0/12	0/2	0/2	0/20	0/20	0/30	0/30	0/194	1/194	0	0.5
	Nymph	38 (29)	0/4	0/4	0/3	0/3	0/1	0/1	0	0	0	0	0	0	0/4	0/4	0	0	0	0	0	0	0	0	0	0	0/5	0/5	0	0	0/3	0/3	0/9	0/9	0/29	0/29	0	0
	Larva	30 (12)	0	0	0	0	0	0	0	0	0/2	0/2	0	0	0	0	0	0	0	0	0	0	0	0	0	0	0/10	0/10	0	0	0	0	0	0	0/12	0/12	0	0
	Subtotal	295 (256)	0/29	0/29	0/32	1/32	0/11	0/11	0/16	0/16	0/5	0/5	0/1	0/1	0/10	0/10	0	0	0/1	0/1	0	0	0	0	0/52	0/52	0/33	0/33	0/2	0/2	0/23	0/23	0/41	0/41	0/256	1/256	0	0.3

*Amblyomma testudinarium*	Nymph	7 (7)	0	0	0	0	0/7	0/7	0	0	0	0	0	0	0	0	0	0	0	0	0	0	0	0	0	0	0	0	0	0	0	0	0	0	0/7	0/7	0	0

*Haemaphysalis flava*	Adult male	6 (6)	0/3	0/3	0	0	0	0	0	0	0	0	0	0	0	0	0	0	0	0	0	0	0	0	0/2	0/2	0/1	0/1	0	0	0	0	0	0	0/6	0/6	0	0
	Adult female	95 (95)	0/25	2/25	0/3	0/3	0/7	0/7	0	0	0/14	0/14	0	0	0	0	0	0	0	0	0/5	0/5	0	0	0/17	0/17	0/15	0/15	0/10	0/10	0/1	0/1	0/3	0/3	0/95	2/95	0	2.1
	Nymph	16 (15)	0/5	0/5	0/3	0/3	0/1	0/1	0	0	0	0	0	0	0	0	0	0	0	0	0/12	0/12	0	0	0/1	0/1	0	0	0	0	0	0	0/5	1/5	0/15	1/15	0	6.3
	Larva	3 (2)	0/1	1/1	0/1	0/1	0	0	0	0	0	0	0	0	0	0	0	0	0	0	0	0	0	0	0	0	0	0	0	0	0	0	0	0	0/2	1/2	0	33.3
	Subtotal	120 (118)	0/34	3/34	0/7	0/7	0/8	0/8	0	0	0/14	0/14	0	0	0	0	0	0	0	0	0/17	0/17	0	0	0/20	0/20	0/16	0/16	0/10	0/10	0/1	0/1	0/8	1/8	0/118	4/118	0	3.3

*Haemaphysalis longicornis*	Adult male	75 (38)	0/5	0/5	0/1	0/1	0	0	0	0	0	0	0	0	0	0	0/8	0/8	0	0	0	0-	0	0	1/9	0/9	0/8	4/8	0/3	0/3	0	0	0/4	0/4	1/38	4/38	1.3	5.3
	Adult female	1236 (1108)	0/127	6/127	0/290	6/290	0/49	3/49	0/1	0/1	0/12	4/12	0/18	2/18	0/11	0/11	0/23	0/23	0/8	0/8	0	0	0/3	0/3	0/110	2/110	1/217	3/217	0/79	0/79	0/48	1/48	0/107	0/107	1/1108	27/1108	0.1	2.2
	Nymph	840 (467)	0/45	1/45	0/77	0/77	1/42	4/42	0/7	0/7	0/9	1/9	0/23	3/23	0	0	0/4	0/4	0/5	0/5	0	0	0/3	0/3	0/2	0/2	5/110	1/110	0/23	0/23	0/6	0/6	0/99	0/99	6/467	10/467	0.7	1.2
	Larva	174 (78)	0/12	7/12	0/23	0/23	0/9	4/9	0	0	0/10	0/10	0/10	2/10	0	0	0/2	0/2	0	0	0	0	0	0	0	0	0/2	0/2	0/1	0/1	0	0	0/9	1/9	0/78	14/78	0	8.0
	Subtotal	2325 (1691)	0/189	14/189	0/391	6/391	1/100	11/100	0/8	0/8	0/31	5/31	0/51	7/51	0/11	0/11	0/37	0/37	0/13	0/13	0	0	0/6	0/6	1/121	2/121	6/337	8/337	0/106	0/106	0/54	1/54	0/219	1/219	8/1691	55/1691	0.3	2.4

Total		2747 (2072)	0/252	17/252	0/430	7/430	1/126	11/126	0/24	0/24	0/50	5/50	0/52	7/52	0/21	0/21	0/37	0/37	0/14	0/14	0/17	0/17	0/6	0/6	1/193	2/193	6/386	8/386	0/118	0/118	0/78	0/78	0/268	2/268	8/2072	60/2072	0.3	2.2

Abbreviations: CLB, *Coxiella*-like bacteria; CO, *Coxiella burnetti*.

^a^16 regions in South Korea: Gangwon (GW), Gyeonggi (GG), Gyeongnam (GN), Gyeongbuk (GB), Gwangju (GJ), Daejeon (DJ), Busan (BS), Seoul (SU), Sejong (SJ), Ulsan (US), Incheon (IC), Jeonnam (JN), Jeonbuk (JB), Jeju Island (JJ), Chungnam (CN), and Chungbuk (CB).

^b^MIR (minimum infection rate) is calculated as the number of positive tick pools divided by the total number of ticks tested, multiplied by 100.

**Table 3:** Monthly detection of *Coxiella*-like bacteria and *Coxiella burnetii* by tick species and developmental stage, including minimum infection rates, from March to December 2024.

Tick		Ticks tested (pools)	Number of positive ticks and pools per month																						MIR^a^	
			March		April		May		June		July		August		September		October		November		December		Total			
			CLB	CO	CLB	CO	CLB	CO	CLB	CO	CLB	CO	CLB	CO	CLB	CO	CLB	CO	CLB	CO	CLB	CO	CLB	CO	CLB	CO
*Ixodes nipponensis*	Adult male	33 (21)	0	0	0/9	0/9	0	0	0	0	0/1	0/1	0	0	0	0	0/5	0/5	0/6	0/6	0	0	0/21	0/21	0	0
	Adult female	194 (194)	0	0	0/43	1/43	0/17	0/17	0/1	0/1	0/5	0/5	0/1	0/1	0	0	0/66	0/66	0/53	0/53	0/8	0/8	0/194	1/194	0	0.5
	Nymph	38 (29)	0	0	0/15	0/15	0/10	0/10	0/2	0/2	0	0	0	0	0	0	0/1	0/1	0/1	0/1	0	0	0/29	0/29	0	0
	Larva	30 (12)	0	0	0/2	0/2	0/10	0/10	0	0	0	0	0	0	0	0	0	0	0	0	0	0	0/12	0/12	0	0
	Subtotal	295 (256)	0	0	0/69	1/69	0/37	0/37	0/3	0/3	0/6	0/6	0/1	0/1	0	0	0/72	0/72	0/60	0/60	0/8	0/8	0/256	1/256	0	0.3

*Amblyomma testudinarium*	Nymph	7 (7)	0	0	0/7	0/7	0	0	0	0	0	0	0	0	0	0	0	0	0	0	0	0	0/7	0/7	0	0

*Haemaphysalis flava*	Adult male	6 (6)	0	0	0/4	0/4	0/1	0/1	0	0	0	0	0	0	0	0	0/1	0/1	0	0	0	0	0/6	0/6	0	0
	Adult female	95 (95)	0	0	0/20	2/20	0/3	0/3	0/1	0/1	0	0	0	0	0	0	0/63	0/63	0/8	0/8	0	0	0/95	2/95	0	2.1
	Nymph	16 (15)	0	0	0/6	1/6	0/4	0/4	0	0	0	0	0	0	0/1	0/1	0/2	0/2	0/2	0/2	0	0	0/15	1/15	0	6.3
	Larva	3 (2)	0	0	0	0	0	0	0	0	0	0	0/1	1/1	0	0	0/1	0/1	0	0	0	0	0/2	1/2	0	33.3
	Subtotal	120 (118)	0	0	0/30	3/30	0/8	0/8	0/1	0/1	0	0	0/1	1/1	0/1	0/1	0/67	0/67	0/10	0/10	0	0	0/118	4/118	0	3.3

*Haemaphysalis longicornis*	Adult male	75 (38)	0	0	1/16	4/16	0	0	0/2	0/2	0/11	0/11	0	0	0/8	0/8	0/1	0/1	0	0	0	0	1/38	4/38	1.3	5.3
	Adult female	1236 (1108)	0/2	0/2	1/126	2/126	0/108	3/108	0/407	3/407	0/305	1/305	0/104	15/104	0/12	3/12	0/28	0/28	0/15	0/15	0/1	0/1	1/1108	27/1108	0.1	2.2
	Nymph	840 (467)	0/3	0/3	6/112	5/112	0/93	0/93	0/160	0/160	0/65	0/65	0/10	5/10	0/2	0/2	0/9	0/9	0/10	0/10	0/3	0/3	6/467	10/467	0.7	1.2
	Larva	174 (78)	0	0	0/4	0/4	0	0	0/1	01	0/1	0/1	0/37	12/37	0/9	2/9	0/19	0/19	0/7	0/7	0	0	0/78	14/78	0	8.0
	Subtotal	2325 (1691)	0/5	0/5	8/258	11/258	0/201	3/201	0/570	3/570	0/382	1/382	0/151	32/151	0/31	5/31	0/57	0/57	0/32	0/32	0/4	0/4	8/1691	55/1691	0.3	2.4

Total		2747 (2072)	0/5	0/5	8/364	15/364	0/246	3/246	0/574	3/574	0/388	1/388	0/153	33/153	0/32	5/32	0/196	0/196	0/102	0/102	0/12	0/12	8/2072	60/2072	0.3	2.2

Abbreviations: CLB, *Coxiella*-like bacteria; CO, *Coxiella burnetti*.

^a^MIR (minimum infection rate) was calculated as the number of positive tick pools divided by the total number of ticks tested, multiplied by 100.

**Table 4:** Detection of *Coxiella*-like bacteria and *Coxiella burnetii* by tick species, developmental stage, wild animal host, and minimum infection rates.

Tick		Tested tick (pool)	Number of positive tick pools/number of pools tested per animal host																				MIR^a^	
			Korean water deer		Hedgehog		Raccoon dog		Roe deer		Mountain rabbit		Badger		Weasel		Red squirrel		Formosan sambar deer		Total			
			CLB	CO	CLB	CO	CLB	CO	CLB	CO	CLB	CO	CLB	CO	CLB	CO	CLB	CO	CLB	CO	CLB	CO	CLB	CO
*Ixodes nipponensis*	Adult male	33 (21)	0/20	0/20	0	0	0	0	0	0	0	0	0/1	0/1	0	0	0	0	0	0	0/21	0/21	0	0
	Adult female	194 (194)	0/177	1/177	0	0	0/11	0/11	0/4	0/4	0	0	0/2	0/2	0	0	0	0	0	0	0/194	1/194	0	0.5
	Nymph	38 (29)	0/23	0/23	0	0	0/3	0/3	0/1	0/1	0	0	0/2	0/2	0	0	0	0	0	0	0/29	0/29	0	0
	Larva	30 (12)	0/12	0/12	0	0	0	0	0	0	0	0	0	0	0	0	0	0	0	0	0/12	0/12	0	0
	Subtotal	295 (256)	0/232	1/232	0	0	0/14	0/14	0/5	0/5	0	0	0/5	0/5	0	0	0	0	0	0	0/256	1/256	0	0.3

*Amblyomma testudinarium*	Nymph	7 (7)	0/7	0/7	0	0	0	0	0	0	0	0	0	0	0	0	0	0	0	0	0/7	0/7	0	0

*Haemaphysalis flava*	Adult male	6 (6)	0/2	0/2	0	0	0/2	0/2	0/1	0/1	0	0	0/1	0/1	0	0	0	0	0	0	0/6	0/6	0	0
	Adult female	95 (95)	0/20	0/20	0	0	0/48	2/48	0/27	0/27	0	0	0	0	0	0	0	0	0	0	0/95	2/95	0	2.1
	Nymph	16 (15)	0/9	1/9	0	0	0/4	0/4	0/2	0/2	0	0	0	0	0	0	0	0	0	0	0/15	1/15	0	6.3
	Larva	3 (2)	0	0	0	0	0/2	1/2	0	0	0	0	0	0	0	0	0	0	0	0	0/2	1/2	0	33.3
	Subtotal	120 (118)	0/31	1/31	0	0	0/56	3/56	0/30	0/30	0	0	0/1	0/1	0	0	0	0	0	0	0/118	4/118	0	3.3

*Haemaphysalis longicornis*	Adult male	75 (38)	1/21	4/21	0	0	0/14	0/14	0/3	0/3	0	0	0	0	0	0	0	0	0	0	1/38	4/38	1.3	5.3
	Adult female	1236 (1108)	1/738	12/738	0/1	0/1	0/192	8/192	0/159	3/159	0	0	0/10	4/10	0	0	0	0	0/8	0/8	1/1108	27/1108	0.1	2.2
	Nymph	840 (467)	6/315	7/315	0/1	0/1	0/87	2/87	0/35	0/35	0	0	0/29	1/29	0	0	0	0	0	0	6/467	10/467	0.7	1.2
	Larva	174 (78)	0/25	5/25	0	0	0/23	5/23	0/2	0/2	0/4	4/4	0	0	0/3	0/3	0/21	0/21	0	0	0/78	14/78	0	8.0
	Subtotal	2325 (1691)	8/1099	28/1099	0/2	0/2	0/316	15/316	0/199	3/199	0/4	4/4	0/39	5/39	0/3	0/3	0/21	0/21	0/8	0/8	8/1691	55/1691	0.3	2.4

Total		2747 (2072)	8/1369	30/1369	0/2	0/2	0/386	18/386	0/234	3/234	0/4	4/4^b^	0/45	5/45	0/3	0/3	0/21	0/21	0/8	0/8	8/2072	60/2072	0.3	2.2

Abbreviations: CLB, *Coxiella*-like bacteria; CO, *Coxiella burnetti*.

^a^MIR (minimum infection rate) was calculated as the number of positive tick pools divided by the total number of ticks tested and multiplied by 100.

^b^A chi-square test indicated significant differences in *C. burnetii* prevalence among host species (*χ*^2^ = 151.340, df = 4, *p* < 0.0001). The highest prevalence was observed in ticks from mountain rabbits (100%, 4/4 pools); however, this result should be interpreted cautiously due to the very small sample size.

## Data Availability

Data supporting the conclusions of this article are included within this article. The newly generated sequences were submitted to the GenBank database under the accession numbers PV682721–PV682755, PV581677–PV581684, and PV582137–PV582196. The datasets used and/or analyzed during the present study are available from the corresponding author upon reasonable request.
